# A New Method for Measuring Bell-Shaped Chest Induced by Impaired Ribcage Muscles in Spinal Muscular Atrophy Children

**DOI:** 10.3389/fneur.2018.00703

**Published:** 2018-09-13

**Authors:** Antonella LoMauro, Paolo Banfi, Chiara Mastella, Katia Alberti, Giovanni Baranello, Andrea Aliverti

**Affiliations:** ^1^Dipartimento di Elettronica, Informazione e Bioingegneria, Politecnico di Milano, Piazza Leonardo Da Vinci, Milan, Italy; ^2^IRCCS Fondazione Don Carlo Gnocchi, Milan, Italy; ^3^Fondazione IRCCS Cà' Granda Ospedale Maggiore Policlinico, SAPRE-UONPIA, Neuropsichiatria dell'Infanzia e dell'Adolescenza, Milan, Italy; ^4^UO Neurologia dello Sviluppo, Fondazione IRCCS Istituto Neurologico Carlo Besta, Milan, Italy

**Keywords:** bell-shaped chest, opto-electronic plethysmography, breathing pattern, paradoxical breathing, SMA, ribcage muscles

## Abstract

The involvement of the respiratory muscular pump makes SMA children prone to frequent hospitalization and morbidity, particularly in type 1. Progressive weakness affects ribcage muscles resulting in bell-shaped chest that was never quantified. The aims of the present work were: (1) to quantify the presence of bell-shaped chest in SMA infants and children and to correlate it with the action of ribcage muscles, assessed by the contribution of pulmonary ribcage to tidal volume (ΔV_RC, p_); (2) to verify if and how the structure of the ribcage and ΔV_RC, p_ change after 1-year in SMA type 2. 91 SMA children were studied in supine position during awake spontaneous breathing: 32 with type 1 (SMA1, median age: 0.8 years), 51 with type 2 (SMA2, 3.7 years), 8 with type 3 (SMA3, 5.4 years) and 20 healthy children (HC, 5.2 years). 14 SMA2 showed negative ΔV_RC, p_ (SMA2px), index of paradoxical inspiratory inward motion. The bell-shaped chest index was defined as the ratio between the distance of the two anterior axillary lines at sternal angle and the distance between the right and left 10th costal cartilage. If this index was < < 1, it indicated bell shape, if ~1 it indicated rectangular shape, while if >> 1 an inverted triangle shape was identified. While the bell-shaped index was similar between HC (0.92) and SMA3 (0.91), it was significantly (*p* < 0.05) reduced in SMA2 (0.81), SMA2px (0.74) and SMA1 (0.73), being similar between the last two. There was a good correlation (Spearman's rank correlation coefficient, ρ = 0.635, *p* < 0.001) between ribcage geometry and ΔV_RC, p_. After 1 year, ΔV_RC, p_ reduced while bell-shaped chest index did not change being significantly lower than HC. The shape of the ribcage was quantified and correlated with the action of ribcage muscles in SMA children. The impaired ribcage muscles function alters the ribcage structure. HC and SMA3 show an almost rectangular ribcage shape, whereas SMA2, SMA2px and SMA1 are characterized by bell-shaped chest. In SMA, therefore, a vicious cycle starts since infancy: the disease progressively affects ribcage muscles resulting in reduced expansion of lung and ribcage that ultimately alters ribcage shape. This puts the respiratory muscles at mechanical disadvantage.

## Introduction

Spinal muscular atrophy (SMA) is an autosomal, recessive, neuromuscular disease characterized by degeneration of α motor neurons in the spinal cord. SMA is caused by a defect in the survival motor neuron (*SMN*) 1 gene; the level of functional SMN protein, which is reported to be related to the clinical severity, depends on the number of copies of a paralogue gene, the *SMN2* gene. SMA is classified as type 0 (prenatal characterized by decreased fetal movements in *utero*), type 1 or Werdnig-Hoffmann disease (very weak infants unable to sit), type 2 (non-ambulant children able to sit unsupported), type 3 (ambulant children) and type 4 (onset in adulthood) (1). The motor neurons degeneration results in progressive weakness and paralysis of both motor and respiratory muscles (2–10). The involvement of the respiratory muscular pump and the consequent respiratory problems increase the risk of hospitalization and morbidity in SMA children. Respiratory failure is the main cause of morbidity or death in SMA children, particularly in type 1 (9–11). The diaphragm is innervated by the phrenic motor neurons that are preserved in SMA (12). The diaphragm, therefore, is relatively spared, while progressive weakness affects ribcage muscles since birth (13, 14). Ribcage muscles are considered accessory muscles and constitute a reserve system to be recruited when the expansion of the ribcage and/or the respiratory effort need to be increased (15). For these reasons, the severe ribcage muscles weakness leads to reduced expansion of pulmonary ribcage and to inefficient cough in SMA. The former results in alveolar hypoventilation, micro-atelectasis and decreased lung distensibility; the latter in poor airway clearance and high risk of pulmonary infections (10, 11, 13, 14, 16). With the recent approval of the first treatment for SMA, and the increasing clinical development of further potential therapeutic approaches, there is a strong need to monitor possible efficacy on respiratory function since infancy. Moreover, longitudinal natural history data to be compared with the effects of new treatments are still limited. Respiratory function can be assessed by invasive or non-invasive techniques during volitional or non-volitional maneuvres (17–19). They can all be used in SMA patients (20, 21), but the assessment of respiratory muscle function in infants/children is limited, due to the objections to using invasive techniques and to the difficulties to obtain reliable volitional maneuvres (22). In contrast to motor function tests, in fact, spirometry and the assessment of respiratory muscle function is not performed in clinical practice in the majority of neonatal and pediatric departments. This is due to difficulties raised by use in this population of respiratory muscle function testing methods developed in adults. Non-invasive non-volitional tests are preferable when infants and children need to be investigated. The analysis during quiet breathing, therefore, must be as much informative as possible. The ventilatory pattern at rest can be easily studied by integrating the flow signal measured at the mouth, but the use of mouthpiece and noseclip could be not well-tolerated by the child. Pulmonary ventilation can alternatively be evaluated by external measurements of chest wall surface motion. These plethysmographic systems use non-ionizing radiations (infrared or structured light) or wire coils insulated within elastic bands (23–25). They also allow thoraco-abdominal respiratory monitoring. Changes in volume of the ribcage and of the abdomen can be respectively inferred from displacements of ribcage muscles and diaphragm. The action of ribcage muscles may, therefore, be quantified by measuring the volume variation of pulmonary ribcage. This in an important additional advantage for SMA children, being the only non-invasive and non-volitional methods to monitor the progression of ribcage muscles weakness. A significantly reduced percentage contribution of pulmonary ribcage to tidal volume is a characteristic feature of SMA, reported in the most severe forms both in adulthood and in childhood (26, 27). Paradoxical inward motion of pulmonary ribcage is systematically present in type I (27). A paradoxical movement occurs when one compartment moves out of phase compared to another one. In SMA type I the pulmonary ribcage moves inward during inspiration rather than outward while the abdomen expands. This can lead to atelectasis of the upper lobes of the lungs and to underdevelopment of the upper chest wall, resulting in bell-shaped chest. In SMA children, the bell-shaped chest is observed and reported but never quantified (9, 11, 14, 16). As far as we know, only one method was proposed to quantify the geometry of ribcage structure. It was applied to children with spastic quadriplegic cerebral palsy and typically developing children. This is the ratio between the transverse diameters of the upper and the lower ribcage measured on chest radiograph (28). It would be interesting to investigate if the altered thoracic structured induced by SMA can be also quantified by non-invasive techniques to avoid unnecessary ionizing radiations.

The primary aim of the present work was to propose a new index based on non-invasive technique able to quantify the presence of bell-shaped chest in type 1, 2, and 3 SMA patients, to compare it with an invasive index, and to find if it correlates with the action of ribcage muscles. Our hypothesis was that the severity-related profile found within the different subtypes of SMA in the expansion of pulmonary ribcage is reflected also in its geometry. Another aim was to verify if and how the structure of the ribcage and the action of ribcage muscles change after 1-year follow-up in a subgroup of SMA type 2.

## Patients and methods

### Patients

In this prospective cross-sectional study, only SMA children younger than 8 years with a genetically proven diagnosis were enrolled. Exclusion criteria were: presence of previous spinal surgery, acute respiratory failure and/or severe airway infections and 24-h mechanical ventilation dependence.

Children with type 1, 2, or 3 SMA were included. All patients received standard care according to the international guidelines (10). Global motor function was assessed by applying functional motor scales: the Children's Hospital of Philadelphia Infant Test for Neuromuscular Disorders (CHOP INTEND) (29, 30) scale for type 1, the Hammersmith functional motor scale expanded (HFMSE) (31) and the Upper Limb Module (ULM) (32) for types 2 and 3.

A control group of healthy children was also included. They were healthy brothers or sisters of SMA patients or relatives and friends of the researches.

A subgroup of SMA type 2 children were also evaluated after 1 year.

The protocol was approved by the Research Ethics Board of Carlo Besta Neurological Research Institute (registration number: CE: 20/2014). At least one parent gave written informed consent in accordance with the Declaration of Helsinki.

### Bell-shaped chest index

**Figure 1:**
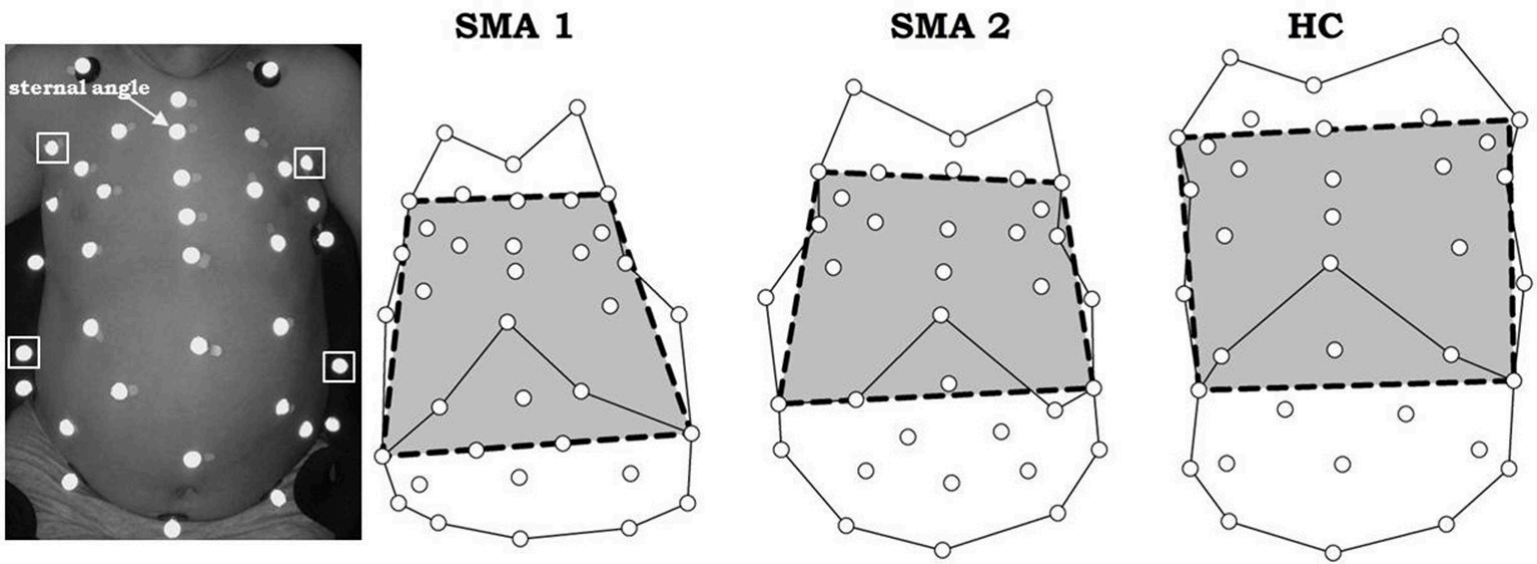
Markers positioning in a SMA type 2 child (left) and markers' projection on the frontal view (right) on three representative subjects: SMA type 1 (SMA1), SMA type 2 (SMA2) and healthy control (HC). The bell-shape chest index is defined as the ratio between the distance of two anterior axillary lines at sternal angle (upper squared markers, left panel) and the distance of the right and left costal cartilage where costal margin reaches its lowest levels (lower squared markers, left panel). Bell-shape chest index < < 1 indicates bell shape. If it was ~1 it indicates rectangular shape, while if >> 1 an inverted triangle shape is identified.

Bell-shaped chest was quantified using the 3D coordinates of passive markers measured by opto-electronic plethysmography (OEP, BTS, Milan, Italy) (23, 33, 34). With the child lying on the bed, 36 markers were placed over his/her anterior trunk surface from clavicles to pubis according to specific anatomical points (27). Using the frontal view of the 3D coordinates, the distance between the two anterior axillary lines at sternal angle (of Louis) and between the right and left 10th costal cartilage, where costal margin reaches its lowest levels, were calculated. The bell-shaped chest index was defined as the ratio between these two distances (Figure 1).

If this index was < < 1, it indicated bell shape, if it was ~1 it indicated rectangular shape, while if >> 1 an inverted triangle shape was identified.

### Upper to lower ribcage ratio

**Figure 2:**
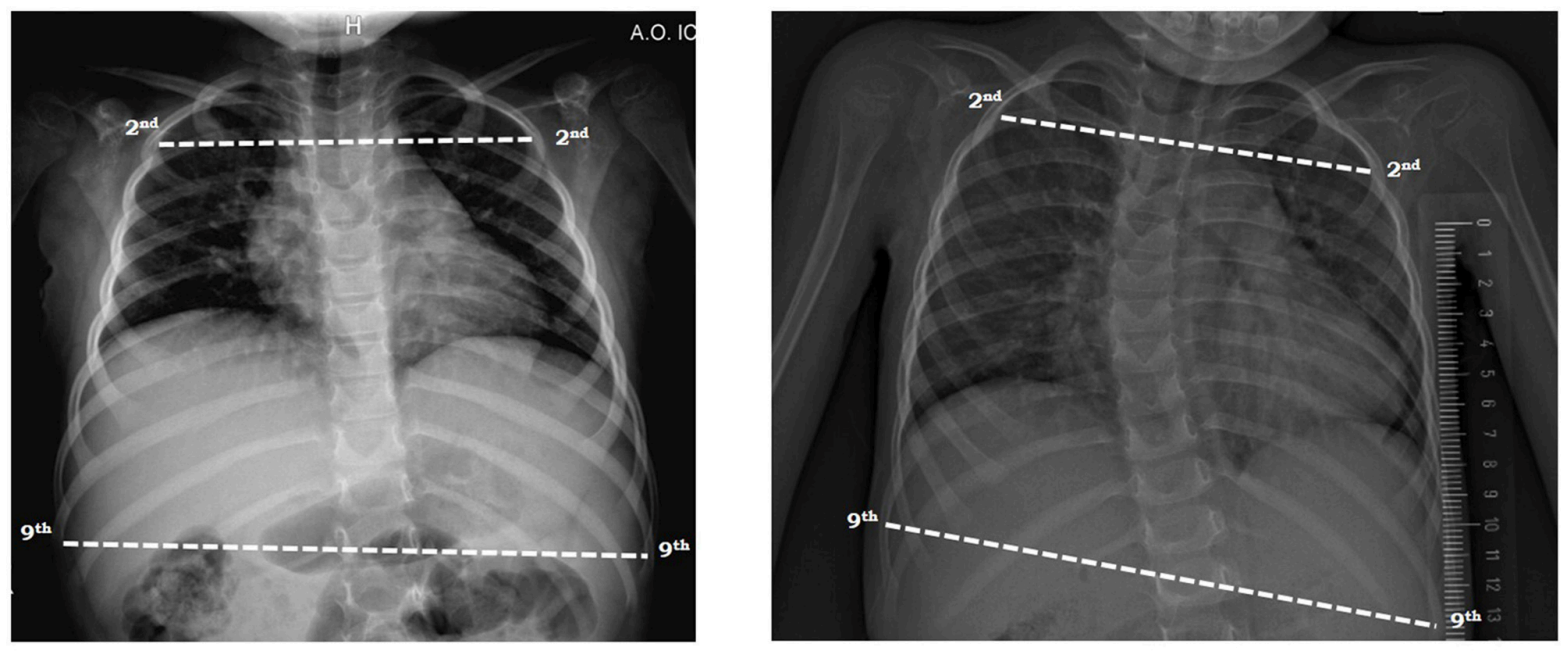
Measurement of the ratio of upper to lower ribcage on antero-posterior view of chest radiograph in a SMA type 2 child with mild **(left)** and severe **(right)** scoliosis in supine position. The lines were drawn from the inner margin of the ribs 2 and 9 on one side to the other side.

The ratio of upper to lower ribcage was computed, respectively between the longest line of the 2nd and the 9th rib (Figure 2) as previously described. Both diameters were calculated on the antero-posterior view of chest radiograph taken in supine position (28).

### Breathing and thoraco-abdominal pattern

Starting from the 3D coordinates and applying the Gauss theorem, it was possible to compute the volume variations enclosed by the passive markers, namely the total chest wall and its three compartments: pulmonary ribcage, abdominal ribcage and abdomen (23). From chest wall volume trace, all the breaths in a stable period of at least 40 s period of awake quite breathing were selected. The entire breathing pattern in terms of minute ventilation, rapid and shallow breathing index and their two components, tidal volume and respiratory rate, were calculated on a breath-by-breath analysis. The percentage contribution to tidal volume of pulmonary ribcage and abdomen were computed as well. The former was defined as the part of the ribcage apposed to the lungs. Its volume was calculated as that enclosed by the clavicles to a line extending transversely at the level of the xiphisternum (35).

### Statistical analysis

The bell-shaped index between the four considered groups (SMA types 1, 2, 3, and healthy children) was compared with a one-way Analysis of Variance (ANOVA) considering disease as the independent factor.

A linear correlation analysis and Pearson product moment correlation coefficient were computed to investigate the linear relationship between bell-shaped chest index and the ratio of upper to lower ribcage.

Spearman rank order correlation was used to assess the relationship between bell-shaped chest index and the percentage contribution of pulmonary ribcage to tidal volume.

Data of the 1-year follow-up study were compared using a one-way ANOVA between healthy children and the subgroup of type 2 SMA children during both visits. If the considered parameter did not pass the normality test, the non-parametric Kruskall-Wallis ANOVA on ranks test was used.

The pairwise multiple comparison procedures were made using the Holm-Sidak method or Dunn's Method, respectively in case of parametric or non-parametric analysis.

All data in table and figures are original and not previously published. They are expressed as median and interquartile range, while in the text the median is represented. Differences were regarded as significant when *p*-values < 0.05.

## Results

### Patients

Ninety-one SMA children were recruited according to the inclusion/exclusion criteria: 32 with type 1 (SMA1, age: 0.82 years), 51 with type 2 (3.74 years), 8 with type 3 (SMA3, 5.40 years); 20 healthy children (HC, 5.20 years) were also assessed.

Anthropometric, motor function, spirometric, cough, breathing and thoraco-abdominal pattern data of SMA children were previously published (27). In summary, SMA1 were younger, whereas type 2, SMA3 and HC were similar in age. The median CHOP INTEND scale score was 22/64; HFMSE scores were 16.5/66 in type 2 and 49/66 in SMA3, while ULM scores were 10/18 and 18/18, respectively. Both HFMSE and ULM scores were significantly lower in type 2 compared to SMA3. Forced vital capacity and peak cough flow were lower in type 2 (0.55 L and 97.2 L/min) than SMA3 (1.34 L and 154.5 L/min). In the same published paper, we demonstrated that a severity-related breathing and thoraco-abdominal pattern was present in SMA. SMA1 children were characterized by rapid and shallow breathing pattern with inward paradoxical movement of pulmonary ribcage. In seated position, also SMA type 2 children showed higher rapid and shallow breathing index with significantly reduced contribution of pulmonary ribcage. SMA3 children were similar to the control group (27).

In addition to these results, we have now added the following new analysis to that dataset.

Consistently with previous studies (36), a HFMSE threshold of 12 was considered in type 2, indicating low- (scores < 12) and high-functioning (scores ≥12) children. The 14 children with HFMSE ≥ 12 were characterized by higher (16.8%) contribution of pulmonary ribcage to tidal volume compared to the 19 children with reduced scores (4.8%, *p* = 0.0018).

### Paradox breathing in SMA type 2

SMA type 2 children were split into two groups: patients showing negative pulmonary ribcage contribution to tidal volume in supine position (SMA2px) and patients with positive pulmonary ribcage contribution (SMA2). SMA2px were 14 and their pulmonary ribcage contribution to tidal volume (−4.9%) was significantly lower (*p* < 0.001) than HC, SMA3 and the 37 SMA2 (12.6%). SMA2px were also characterized by lower pulmonary ribcage contribution to tidal volume in seated position (3.1%), compared to SMA2 (21.7%), SMA3 and HC (*p* < 0.001). The percentage contribution of pulmonary ribcage to tidal volume of SMA2 was lower than SMA3 (*p* = 0.007) and HC (*p* = 0.003) in seated position, while it was similar in supine. Motor functional scales were available in 7 SMA2px and 23 SMA2 children. Although HFMSE scores were lower in SMA2px (8) compared to SMA2 (12), the difference did not reach statistical significance (*p* = 0.08). ULM scores were similar between the two groups (9 and 10, respectively; *p* = 0.550).

### Bell-shaped chest index

**Figure 3:**
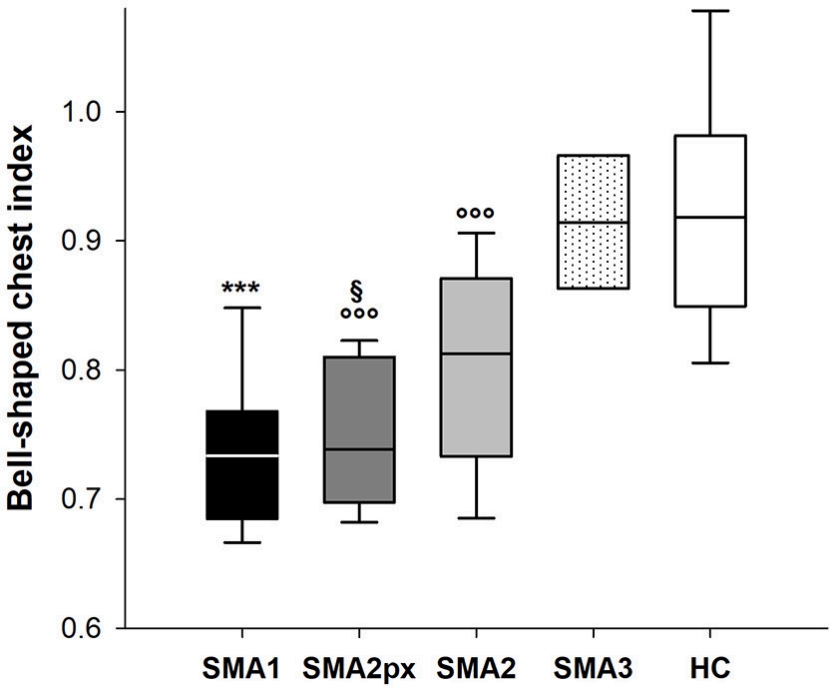
Box-and-whisker plot representing the median (line within the box), the interquartile range (length of the box), the 90 and the 10th percentiles (whiskers above and below the box) of bell-shaped chest index in SMA type 1 (SMA1), type 2 with paradoxical inward motion of pulmonary ribcage (SMA2px), type 2 without paradoxical breathing (SMA2), type 3 (SMA3) and healthy children (HC). ^***^p < 0.001 vs. SMA2, SMA3 and HC; °°°*p* < 0.001 vs. SMA3 and HC; ^§^*p* < 0.05 vs. SMA2.

Figure 3 shows the bell-shaped chest index in the five considered groups. While this index was similar between HC (0.92) and SMA3 (0.91), it was significantly reduced in SMA2 (0.81), SMA2px (0.74) and SMA1 (0.73), being similar between the last two.

**Figure 4:**
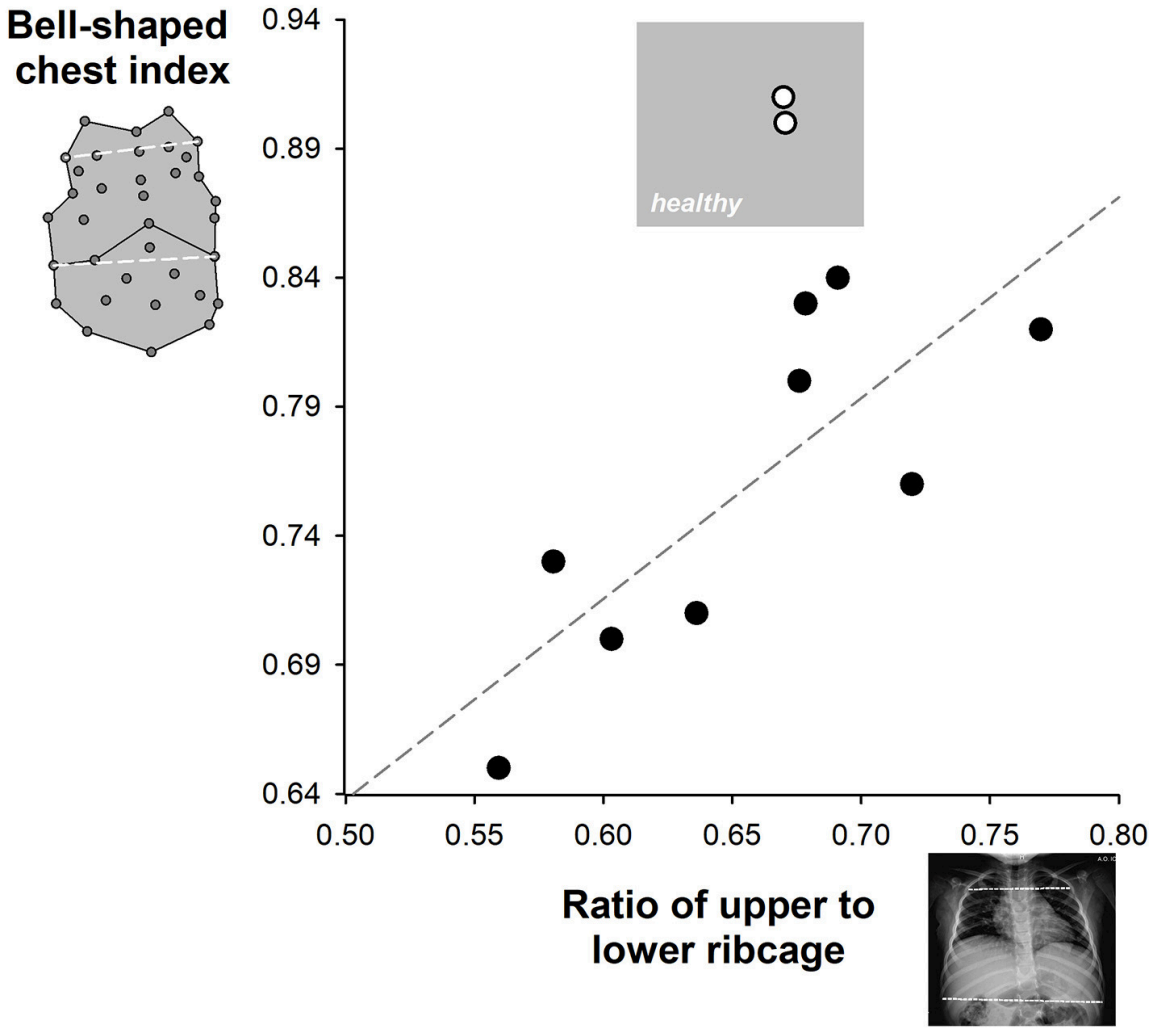
Correlation between the upper to lower ribcage ratio (x-axis) and the bell-shape chest index (y-axis) available in SMA type 2 children. The gray area represents the zone of normal values. The limits of the upper to lower ribcage ratio were obtained from the literature (28). Two SMA type 2 children (white symbols) were characterized by normal values of both indexes. The correlation line was calculated among the remaining nine children (black symbols).

Concomitant antero-posterior view of chest radiograph was available on 11 SMA type 2 who suffered from scoliosis. Their ratio of upper to lower ribcage was 0.68 and it was compared to the corresponding bell-shaped chest index in Figure 4. Both indexes were within normal ranges in two patients who were therefore excluded in the correlation analysis. A good linear correlation was found between the two indexes (coefficient of determination *r*^2^ = 0.635, Pearson correlation coefficient = 0.797; *p* = 0.0101) in the remaining nine children.

**Figure 5:**
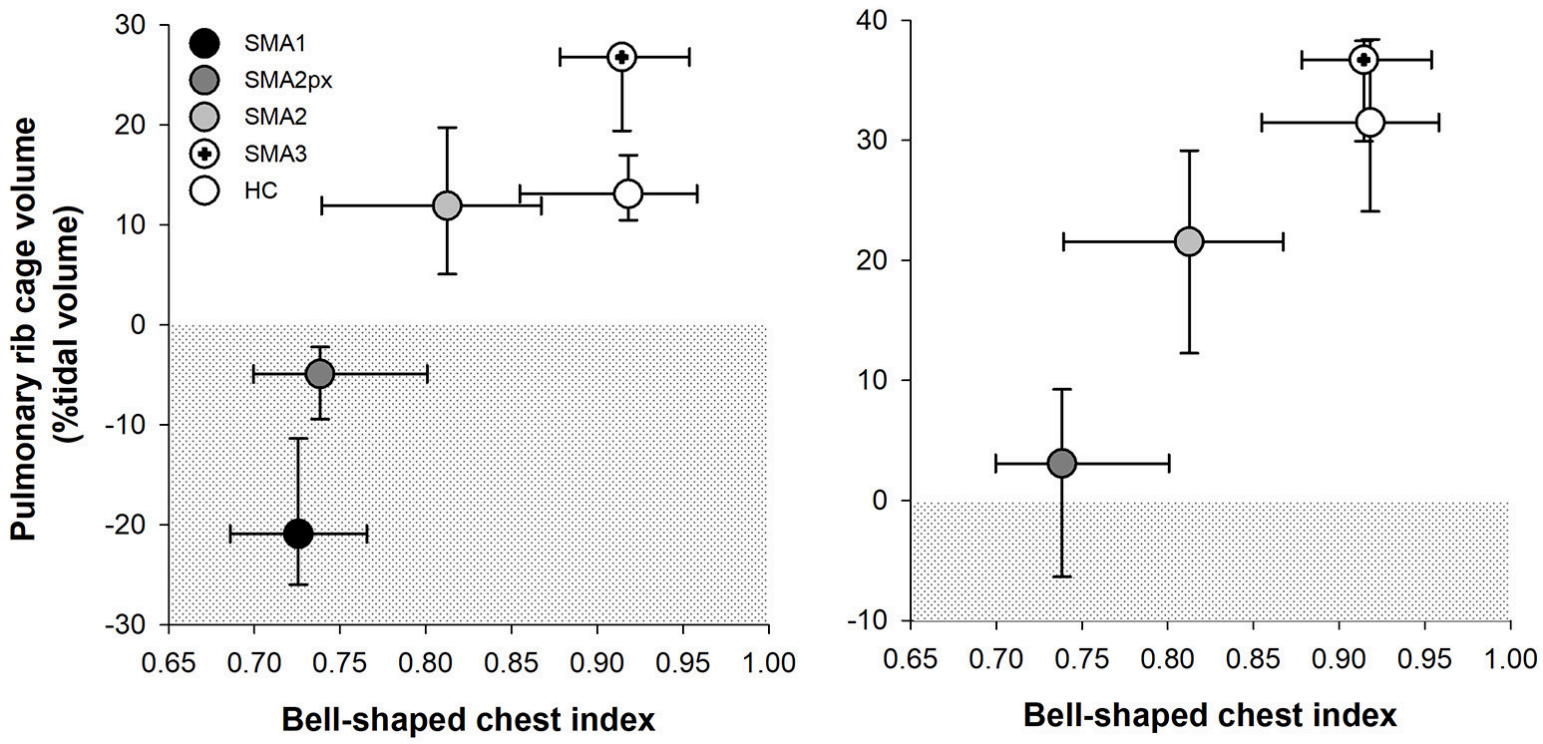
Median and interquartile range (error bars) of bell-shaped chest index (*x axis*) plotted against the median and interquartile range (error bars) of pulmonary ribcage contribution to tidal volume in supine position (*y axis*) in SMA type 1 (SMA1), type 2 with paradoxical inward motion of pulmonary ribcage (SMA2px), type 2 without paradoxical breathing (SMA2), type 3 (SMA3) and healthy children (HC). The dotted area represents the paradoxical inward movement of pulmonary ribcage.

There was a good correlation both in supine (Spearman's rank correlation coefficient, ρ = 0.635, *p* < 0.001) and in seated (ρ = 0.535, *p* < 0.001) position between bell-shaped chest index and the contribution of pulmonary ribcage to tidal volume as shown in Figure 5. On the other hand, bell-shaped chest index did not differ between type 2 children with HFMSE scores >12 (0.81) compared to those with reduced scores (0.77, *p* = 0.249).

### One-year follow-up study in type 2

#### Patients

Twenty-four type 2 children (5 SMA2px and 19 SMA2) were analyzed at 1-year follow-up. Age and height, but not weight, significantly increased after 1 year. Motor function scores were available in 18 children. While HFMSE scores did not change, ULM scores significantly increased by two points after 1 year. No difference was found in forced vital capacity and peak cough flow that were obtained in six patients (Table 1). Information on the use of nocturnal non-invasive ventilation (NIV) was available in 18 children. While none of them used nocturnal NIV at baseline, it was routinely used by 6 out of the 18 children at follow up.

**Table 1:** Median and interquartile range (IQR) of anthropometric, motor function, spirometric, and cough data of the SMA type 2 children with two visits.

	**First visit**		**Second visit**		
	**Median**	**IQR**	**Median**	**IQR**	***p*-value**
*Anthropometry (n = 24)*			*(n = 24)*		
Age (yrs)	3.8	(3.4–4.8)	5.1	(4.5–5.7)	***0.000***
Height (cm)	100.0	(92.0–109.0)	104.0	(100.0–108.1)	***0.002***
Weight (Kg)	14.2	(12.5–17.0)	14.9	(13.3–17.8)	*0.291*
*Motor function (n = 22)*			*(n = 18)*		
HFMSE (/66)	18.2	(12.0–30.5)	16.5	(7.4–24.8)	*0.808*
ULM (/18)	9.0	(6.0–13.0)	11.0	(9.0–14.5)	***0.035***
*Spirometry and cough* (n = 11)			*(n = 6)*	
FVC (L)	0.53	(0.41–0.90)	0.69	(0.59–0.79)	*0.104*
FVC (%pred)^*^	65.4	(52.9–82.8)	73.1	(50.5–91.9)	*0.172*
PCF (L/s)	1.61	(0.93–1.81)	1.48	(1.13–1.72)	*0.458*
PCF (%pred)^**^	51.1	(34.4–59.6)	42.6	(37.5–47.7)	*0.487*
*Chest geometry (n = 21)*			*(n = 23)*		
Bell-shaped chest index	0.78	(0.74–0.88)	0.80	(0.72–0.87)	*0.655*

* *Reference values from Quanjer et al. (37)*.

** *Reference values from Bianchi and Baiardi (38)*.

*The p-values of the difference between the two visits are reported in the last column. The p-values < 0.05 are in bold*.

#### Breathing and thoraco-abdominal pattern

**Figure 6:**
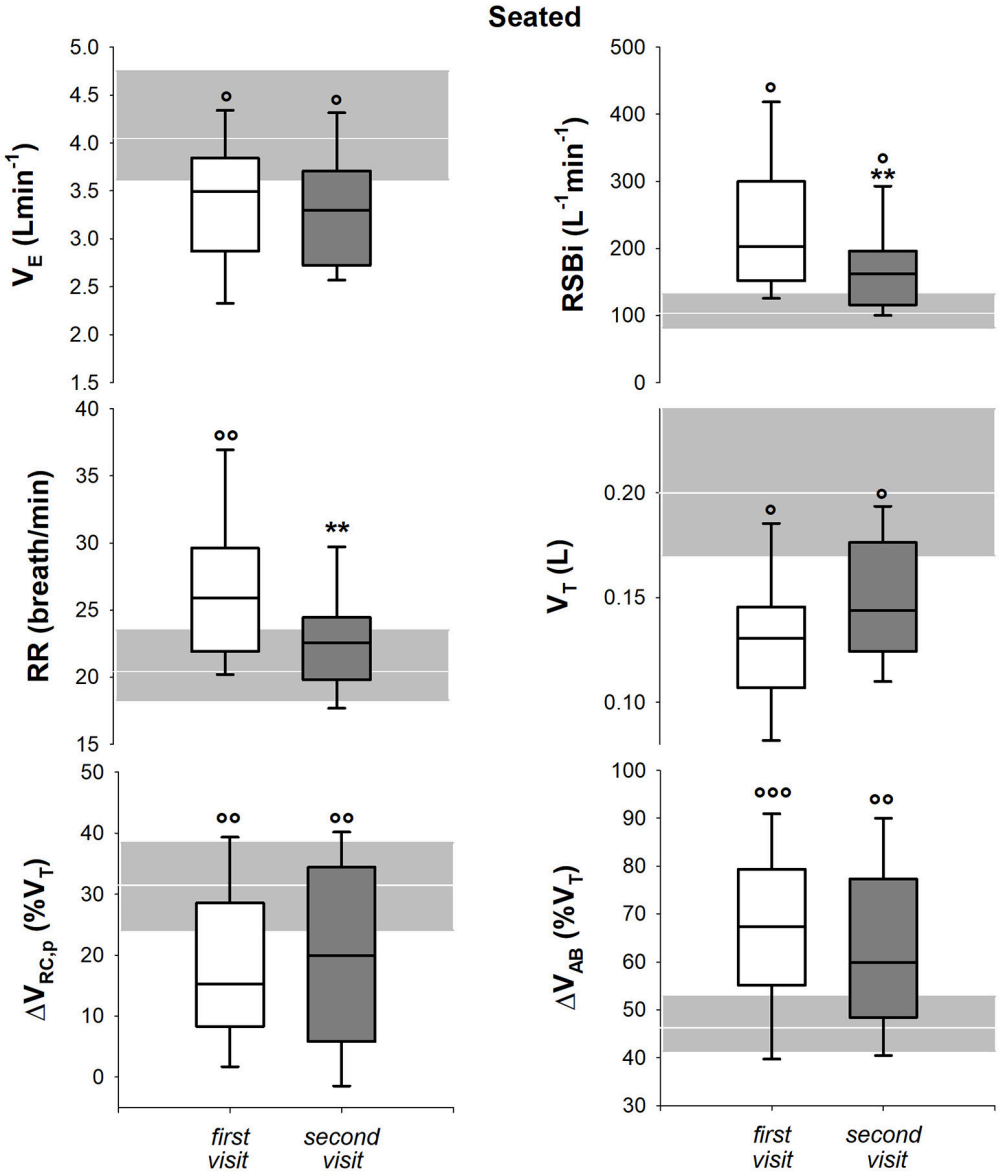
Box-and-whisker plot representing the median (line within the box), the interquartile range (length of the box), the 90 and the 10th percentiles (whiskers above and below the box) of minute ventilation (V_E_, top left), rapid and shallow breathing index (RSBi, top right), respiratory rate (RR, middle left), tidal volume (V_T_, middle right), pulmonary ribcage contribution to tidal volume (ΔV_RC, p_ (%V_T_), bottom left) and abdominal contribution to tidal volume (ΔV_AB_ (%V_T_), bottom right) in seated position in SMA type 2 children at the first visit and 1-year follow-up (second visit). The gray area and the white line represent the interquartile range and the median of the healthy control group. V_E_ = RR^*^V_T_; RSBi = RR/V_T_. ^**^p < 0.01 vs. first visit; °, °°, °°°*p* < 0.05, 0.01, 0.001 vs. healthy control group.

**Figure 7:**
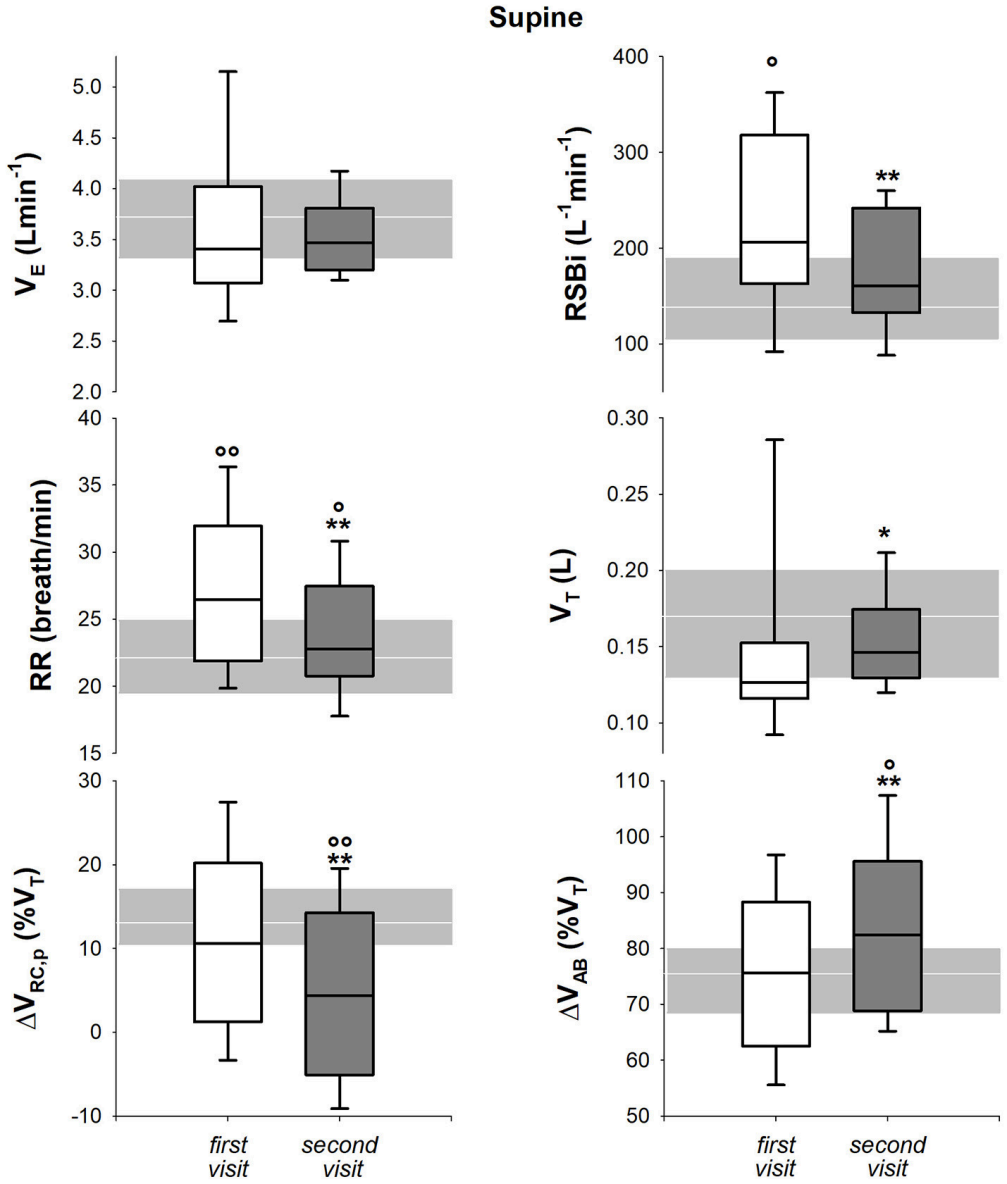
Box-and-whisker plot representing the median (line within the box), the interquartile range (length of the box), the 90 and the 10th percentiles (whiskers above and below the box) of minute ventilation (V_E_, top left), rapid and shallow breathing index (RSBi, top right), respiratory rate (RR, middle left), tidal volume (V_T_, middle right), pulmonary ribcage contribution to tidal volume (ΔV_RC, p_ (%V_T_), bottom left) and abdominal contribution to tidal volume (ΔV_AB_ (%V_T_), bottom right) in supine position in SMA type 2 children at the first visit and 1-year follow-up (second visit). The gray area and the white line represent the interquartile range and the median of the healthy control group. V_E_ = RR^*^V_T_; RSBi = RR/V_T_. ^*, **^p < 0.05, 0.01 vs. first visit; °, °°*p* < 0.05, 0.01 vs. healthy control group.

In seated position, only respiratory rate and rapid and shallow breathing index decreased after 1 year. All the parameters, with the exception of 1-year follow-up respiratory rate, were significantly lower than healthy children both at baseline and at follow up (Figure 6). In supine position, all the parameters but minute ventilation changed after 1 year. Rapid and shallow breathing index, respiratory rate and percentage contribution of pulmonary ribcage to tidal volume decreased. Tidal volume and abdominal percentage contribution to tidal volume increased. In both visits, minute ventilation and tidal volume were similar to the control group whereas respiratory rate was higher. Rapid and shallow breathing index was higher than healthy children at baseline, but it became similar after 1 year. On the contrary, the thoraco-abdominal contribution to tidal volume was similar to controls at baseline, but it showed a reduction in pulmonary ribcage and a concomitant increase in abdominal contribution (Figure 7) at 1 year follow up. After 1 year, four children showed negative pulmonary ribcage contribution to tidal volume in supine position in addition to the five at baseline. Children under NIV were characterized by negative pulmonary ribcage contribution to tidal volume in supine position (−5.6%), being significantly lower than children not under NIV (11.2%, *p* = 0.00978). In seated position, all children under NIV but one had positive pulmonary ribcage contribution (4.2%) that tended to be lower compared to children not under NIV (20.4%) although not statistically significantly (*p* = 0.135).

#### Bell-shaped chest index

Although bell-shaped chest index did not change between the first and the second visit (Table 1), it was significantly lower than healthy children in both visits (*p* = 0.0002 at baseline and *p* = 0.0003 at 1-year follow-up). In the five SMA2px, bell-shaped chest index tended to be lower than SMA2 (0.744 and 0.819, respectively) at baseline, even if not significantly (*p* = 0.12). On the other hand, after 1 year it became significantly lower between the nine SMA2px (0.755) and the remaining 13 SMA2 children (0.841, *p* = 0.021). Bell-shaped chest index was 0.73 in NIV children and 0.86 in those not under NIV (*p* = 0.05).

## Discussion

For the very first time, the action of ribcage muscles and the shape of ribcage were quantified and correlated in children affected by SMA. The impaired ribcage muscles function, induced by the disease, alters the ribcage structure of these children resulting in bell-shaped chest. Pulmonary ribcage, therefore, does not grow properly in particular when paradoxical breathing is present. This was obvious not only in SMA 1, but also in a proportion of children with SMA 2. After 1 year, the impairment of ribcage muscles in SMA type 2 gets worse so that the number of children with thoraco-abdominal paradoxical breathing in supine position increases.

In the present paper, we have focused our analysis on the thoracic wall. In SMA, in fact, it is extremely important to study the thorax, because its muscles are the most affected respiratory ones, and its shape is the scaffolding structure for the respiratory system. The impairment of the thoracic wall is thus essential in the management of SMA and longitudinal data on the evolution of this aspect are still limited.

The main roles of ribcage muscles are to elevate the ribs, to expand the pulmonary ribcage and to prevent its inward drawn secondary to the contraction of the diaphragm. The action of ribcage muscles is minimal during quiet breathing, particularly in supine position, but they increase and enhance their activity when ventilation and/or the respiratory mechanical load increase (39).

We have previously shown that in the two most severe forms of SMA the breathing pattern is shifted toward the abdomen, therefore toward the diaphragm, and it is characterized by poor action of ribcage muscles since infancy and childhood (27).

The most important consequences of ribcage muscles weakness are cough inefficiency and the difficulty to take deep breaths, particularly during sleep, that would help to maintain normal oxygen and carbon dioxide levels. Consequently, the lungs are not fully inflated and this can lead to underdevelopment of lung tissue, widespread microatelectasis and decreased distensibility that may contribute to restrictive lung function in adulthood. Normal expansion of the growing lungs is very important because it helps the ribcage to develop and maintain a normal shape. It is important to consider the shape of the ribcage as it is the scaffolding structure that provides the best mechanical advantage for lungs and ribcage muscles to work optimally. Deviation from the ideal ribcage structure can result in respiratory dysfunction.

The geometry of the ribcage can be obtained from chest radiographs (28, 40), semi-landmark methods on computed tomography (41) and by opto-electronic systems for motion analysis based on infrared light (33, 34).

Only one group tried to quantify the reduced growth of upper ribcage by measuring the percentage ratio of ribcage apex and base diameters on antero-posterior view of a chest *x*-ray. This method was applied to children with severe spastic quadriplegic cerebral palsy without scoliosis and healthy peers. The ribcage ratio was lower in the former (28). This index seems easily applicable and useful in SMA children, however it has some intrinsic limitations: (1) it requires ionizing radiations, therefore it does not seem suitable for longitudinal studies to monitor the progression of the disease in infancy; (2) chest *x*-ray is not one of the recommended test in the management of SMA (9, 10). Because radiation sensitivity in children is considerably higher than in adults, at least by a factor 10 (42), it is better to avoid unnecessary exosition. Maybe chest *x*-ray can be available in case of pneumonia or scoliosis; (3) the index was defined in the absence of scoliosis, that, in turn is a common feature in SMA children, particularly type 2.

In our clinical database, for example, chest *x-ray* were available only in 11 SMA type 2 children who suffered from scoliosis. On the other hand, the 3D coordinates of the markers obtained by opto-electronic plethysmography were available in all the children. Inspired by the proposed ribcage ratio, without the use of ionizing radiation we have introduced the bell-shpaed chest index to quantify and characterize the shape of the ribcage in quite a high number of children affected by the three main forms of SMA.

Although the two indexes showed a strongly linear correlation, their results may apparently seem conflictual. Surprisingly, the ribcage ratio was higher than the reported healthy range in all but two SMA children, while important differences emerged in the analysis of the bell-shaped index. Similarly to healthy peers, the thorax in type 3 SMA shows an almost rectangular shape, whereas in type 2 and 1 the bell-shaped index was significantly lower. This gives the appearance of a bell-shaped chest because the top of the ribcage, is narrow and the bottom of the thorax is much wider.

This discrepancy may be explained by different considerations: (1) chest *x-ray* was available in a small number of children. Moreover, they were all affected by SMA type 2. No data, therefore, are available to understand if the ribcage ratio is able to distinguish among the different forms of the disease; (2) the ribcage ratio was adapted to the presence of scoliosis. The possible rotation of the facet joint or spinous process secondary to spinal deformity may have influenced the final result; (3) the different race of the control group for the ribcage ratio. Spirometry slightly differs between Caucasian and Asian population (37); we can speculate that race may affect also ribcage shape; (4) the ribcage ratio and the bell-shaped index are referred to two different, but strongly correlated, structures: the ribcage and the thoracic wall, respectively. The latter, in fact, is made up by the ribcage together with the skin, fat and associated fascia and muscles. The bell-shaped chest index, therefore, takes into account also the condition of ribcage muscles. For this reason, the bell-shaped chest index seems to be more sensitive to detect the altered chest geometry. We expect ribcage muscles to be the major determinant of bell-shaped, because in SMA they are not strong enough to pull the top of the ribcage out against the pulling down action of the diaphragm.

The bell-shaped chest index becomes worse when paradoxical inward thoracic motion is present. While paradoxical breathing seems systematic in the worst form, as all but one SMA1 children showed it, it was observed in one third of SMA2 children. When thoraco-abdominal paradoxical breathing is present in type 2, it is associated to worse bell-shaped chest index and worse HFMSE scores, although not still statistically significant maybe because of the low number of the available values. The inward paradoxical expansion of the upper part of the ribcage in supine position, therefore, seems to be a discriminant factor in the severity of clinical condition of type 2 SMA children. It was also the only parameter that gets worse over 12 months in SMA2.

The HFMSE scores, forced vital capacity and peak cough did not change over time; we only observed an increase by two points on the ULM scale. This was probably related to maturation and/or sensitivity to change, which is consistent with the recent revision of the scale. The reduction in respiratory rate found both in supine and seated position can be expected because it normally decreases with growth until the age of 13 years when it reaches adult values. Conversely, tidal volume is reported to increase with age (43). In our cohort of SMA2, tidal volume increases in both postures, but it reaches statistically significance only in supine position. As a result, rapid and shallow breathing index reduces in both postures. The thoraco-abdominal contributions to tidal volume are invariant with age (44, 45). While in seated position there were no significant changes over 12 months, in supine position the pulmonary ribcage contribution in SMA2 significantly decreased with concomitant increase in abdominal contribution due to the reduced contribution of ribcage muscles and the increase of diaphragm activity. The percentage of children with paradoxical breathing in supine position increases from 21 to 37.5% in 1 year and it is accompanied by worse bell-shaped chest index.

In SMA, therefore, a vicious cycle starts since infancy: the disease *per se* progressively affects ribcage muscles resulting in reduced expansion of lung and ribcage that ultimately affects chest development. The altered ribcage shape put all the respiratory muscles at mechanical disadvantage: diaphragmatic fatigue was reported in some type 2 SMA children after the age of 10 (21). This results in thoraco-abdominal asynchrony with consequent rapid and shallow breathing pattern. Global motor function of SMA2 children with paradoxical breathing, and therefore with severe bell-shaped chest, tends to be lower. Interestingly, children under NIV showed significantly lower negative pulmonary ribcage contribution to tidal volume in supine position and lower bell-shaped chest index than children not under NIV. Further studies should be performed to assess if long-term use of NIV is able to prevent this evolution.

The index that we have proposed in the present study can be considered a good surrogate of the action of ribcage muscles action, as it correlated with the presence of paradoxical breathing and with the degree of severity of SMA. It is a simple easy-to-obtain non-invasive method that does not require the cooperation of the child and that can be performed in every setting from every professional dealing with children with SMA. Although we have used opto-electronic plethysmography, the bell-shaped chest index consists of the measure of two distances, according to specific anatomical points, that can be obtained with a measuring tape. Dedicated tests should be focused to understand its potentiality in different applications and if it can be easily reproducible by others. This is one important strength of the study. Although our results were somewhat expected because weakness of intercostal muscles is a characteristic feature of SMA, paradoxical breathing and bell-shaped chest are observed, for the very first time they have been quantified and proved on a relatively high number of children affected by the three forms of SMA. This assessment could be a reliable method to test efficacy of new emerging treatments on respiratory function over time. Having included homogenous groups in terms of severity of the disease and age and having compared them with healthy kids is another strength of the study. A limitation of the study is the weak part of the validation of the bell-chest index, particularly due to the lack of a gold standard. A second limitation of the study is the lack of follow-up data on a period longer than 1 year and on the most severe type 1 form, but these data showed the feasibility of OEP to be used also in longitudinal study.

We believe that the proposed non-invasive, non-volitional method showed how strongly informative is the analysis of thoracic geometry and of quiet breathing. It can be applied to all children and it helps monitoring the progression of SMA and/or quantifying the potential benefit of recently approved treatments or in clinical trials.

## Conclusions

According to the severity of SMA, the impaired ribcage muscles function, induced by the disease, alters the ribcage structure of these children resulting in bell-shaped chest. The presence of paradoxical breathing in supine position in SMA type 2 is associated to bell-shaped chest index similar to SMA type 1 and worse HFMSE scores. We have proposed a simple and non-invasive index to quantify the bell-shaped chest that can be performed in every laboratory and can be a good surrogate of the action of ribcage muscles.

## Author contributions

AL conception and design, acquisition of data, analysis and interpretation, statistical analysis, and drafting of the manuscript for important intellectual content. CM, KA, PB, and GB clinical evaluation. AL, CM, KA, PB, GB, and AA final approval of the version to be published.

### Conflict of interest statement

The authors declare that the research was conducted in the absence of any commercial or financial relationships that could be construed as a potential conflict of interest.
